# Assessment of Spectral Doppler in Preclinical Ultrasound Using a Small-Size Rotating Phantom

**DOI:** 10.1016/j.ultrasmedbio.2013.03.013

**Published:** 2013-08

**Authors:** Xin Yang, Chao Sun, Tom Anderson, Carmel M. Moran, Patrick W.F. Hadoke, Gillian A. Gray, Peter R. Hoskins

**Affiliations:** British Heart Foundation Centre for Cardiovascular Science, University of Edinburgh, Edinburgh, UK

**Keywords:** Blood velocity, Doppler ultrasound, High-frequency ultrasound, Doppler phantom, Preclinical ultrasound

## Abstract

Preclinical ultrasound scanners are used to measure blood flow in small animals, but the potential errors in blood velocity measurements have not been quantified. This investigation rectifies this omission through the design and use of phantoms and evaluation of measurement errors for a preclinical ultrasound system (Vevo 770, Visualsonics, Toronto, ON, Canada). A ray model of geometric spectral broadening was used to predict velocity errors. A small-scale rotating phantom, made from tissue-mimicking material, was developed. True and Doppler-measured maximum velocities of the moving targets were compared over a range of angles from 10° to 80°. Results indicate that the maximum velocity was overestimated by up to 158% by spectral Doppler. There was good agreement (<10%) between theoretical velocity errors and measured errors for beam-target angles of 50°–80°. However, for angles of 10°–40°, the agreement was not as good (>50%). The phantom is capable of validating the performance of blood velocity measurement in preclinical ultrasound.

## Introduction

Imaging modalities, including magnetic resonance imaging (MRI) and ultrasound, are finding increasing application in preclinical research (Foster et al. 2000, 2011; Gray et al. 2013; Greco et al. 2012; Moran et al. 2013). These imaging modalities enable longitudinal studies to be performed, increasing the statistical power of investigations with a consequent reduction in the number of animals required. The term *preclinical* generally refers to biomedical research involving the use of small animals, such as mice, rats and increasingly zebra fish, in the development of new diagnostic methods and therapies before trials in humans (*i.e.,* “clinical” research). Key vessels of interest in preclinical work are the aorta, carotid and femoral arteries. These have typical diameters of 0.3–2.0 mm in the rat and 0.15–1.0 mm in the mouse. The typical axial resolution is 50–100 μm for preclinical ultrasound, and in practice, good-quality images of arteries can be obtained in mice and rats. The improvement in spatial resolution of preclinical compared with clinical ultrasound is achieved through the use of higher frequencies. Preclinical ultrasound systems have transmit frequencies in the range 20–50 MHz, compared with 3–12 MHz for clinical ultrasound.

Measurement of blood velocity is performed using the Doppler effect, both in the microcirculation and in major arteries (Christopher et al. 1997; Goertz et al. 2003). Blood velocity has been used as a surrogate for volumetric flow (Bonnin et al. 2008; Hartley et al. 2008; Ishikawa et al. 2003; Li et al. 2008) and for estimation of the degree of stenosis in models of atherosclerosis (Ni et al. 2008).

Although there has been consideration of velocity measurement errors in clinical ultrasound, there is a lack of information on preclinical ultrasound systems. In clinical practice, blood velocity is commonly measured using the maximum Doppler frequency shift. Commercial ultrasound systems overestimate blood velocity, typically by 0%–60%, but this can increase to more than 100% when the Doppler angle approaches 80°–90° (Hoskins 1996, 1999; Hoskins et al. 1991). Typical errors generated in routine clinical practice could lead to mis-categorization of patients for carotid surgery (Hoskins 1996).

According to the Doppler equation in its simplest form, a single velocity at any instant in time should give rise to a single Doppler frequency shift at that instant. In practice, a single velocity may give rise to a range of Doppler frequencies. This phenomenon is referred to as *spectral broadening* and may give rise to errors in the blood velocity when estimated from the maximum Doppler frequency. There are a number of sources of spectral broadening (*e.g.,* see Evans and McDicken 2000; Hoskins 2002). The five principal types are:

• *Non-stationarity broadening* is associated with variations in velocity during the sampling time (Fish 1991). This is thought to be relevant mainly during times when the velocity values are changing rapidly, such as in early systole.
• *Velocity gradient broadening* is associated with a range of velocities or directions within the Doppler sample volume. This leads to additional frequencies below the maximum and so, in principle, should not affect maximum Doppler frequency shift.
• *Multi-direction broadening* is associated with a range of velocity directions within the sample volume. This is a significant issue in turbulent flow.
• *Transit time broadening* is associated with the length of time a scatterer remains in the sample volume.
• *Geometric spectral broadening* is associated with the range of angles that the scatterer subtends at the transducer (Censor et al. 1988; Newhouse et al. 1980).

Transit time broadening and geometric spectral broadening had, for a long time, been thought to be equivalent (Newhouse et al. 1980); however, these were shown to be different phenomena by Guidi et al. (2000). For clinical ultrasound systems, it has been found that the maximum Doppler frequency estimation and maximum velocity estimation can be accurately modeled assuming only geometric spectral broadening (Hoskins 1999; Hoskins et al. 1999). This implies that geometric spectral broadening is the main source of error for velocity estimation using clinical ultrasound systems.

In geometric spectral broadening, the finite size of the Doppler aperture means that blood in the sample volume is insonated by a range of angles rather than a single angle. The highest Doppler shift occurs at one extreme edge of the Doppler aperture, whereas in practice, manufacturers perform angle correction with respect to the center of the aperture.

The evaluation of these errors for clinical ultrasound scanners necessitated the development of a range of phantoms involving moving targets, including string and flow phantoms (reviewed in Hoskins 2008). Similar errors are likely to exist for preclinical ultrasound systems, but this is difficult to establish as few Doppler test phantoms have been optimized for preclinical scanners. The aim of this investigation was to develop phantoms for evaluation of Doppler ultrasound-derived velocity values made using preclinical ultrasound systems, with comparison of detected errors with predictions, based on a ray model of geometric spectral broadening.

## Methods

### Theory and simulations

The effect of geometric spectral broadening on velocity error was modeled using a previously published ray model (Hoskins 1999). For an un-steered beam produced from a transducer with a width *D* and focal depth *L*, the maximum Doppler frequency may be described by the equation

(1) $$\partial F_{\max}=\left(2FV/c\right)\left[\cos\left(\theta \right)+\left(D/2L\right)\sin\left(\theta \right)\right]$$

where *F* = transmit frequency; *V* = velocity; *c* = speed of sound; and *θ* = beam-target angle.

Equation (1) assumes that the beam width at the focus is zero. For a finite beam width *w,* we may use the equation

(2) $$\partial F_{\max}=\left(2FV/c\right)\left[\cos\left(\theta \right)+\left(\left(D+w\right)/2L\right)\sin\left(\theta \right)\right]$$

Typical Doppler systems perform conversion from Doppler frequency to velocity with respect to the center of the Doppler aperture, in which case

(3) $$\partial F_{\max}=\left(2FV/c\right)\cos\left(\theta \right)$$

The error *V*_err_ in estimated velocity (*V*_est_) s defined as

(4) $$V_{\text{err}}=\left(V_{\text{est}}-V\right)/V$$

Rearranging eqns (1), (3) and (4) for the zero-width model yields

(5) $$V_{\text{err}}=\left(D/2L\right)\tan\left(\theta \right)$$

and rearranging eqns (2), (3) and (4) for the finite-width ray model yields

(6) $$V_{\text{err}}=\left[\left(D+w\right)/2L\right]\tan\left(\theta \right)$$

For each transducer, eqns (5) and (6) were used to calculate the theoretical error in maximum velocity as a function of angle, using data on aperture size (*D*), focal depth (*L*) and beam width (*w*) provided below.

### Ultrasound scanner and beam width measurement

Ultrasound scanning was performed using a Vevo 770 high-frequency ultrasound system (VisualSonics, Toronto, ON, Canada), which has a range of single-element transducers of different frequency and focal depth. The element in each transducer was circular in shape, with focusing produced by an acoustic lens. It is known that in this arrangement, the region of best resolution occurs at the focal zone. It is not possible to use electronic focusing to improve beam characteristics outside of the focal zone as this requires the use of multi-element arrays. The single element was contained in a plastic housing with an acoustic window. The element was mechanically swept to and fro to produce a real-time image. Further details of beam forming in single elements and of mechanically swept real-time transducers can be found in standard textbooks (*e.g.,* Hoskins et al. 2010; Wells 1977).

We tested five transducers with the parameters given in Table 1. To determine the predicted errors introduced by geometric spectral broadening, the beam-width parameter is needed. A 0.2-mm membrane hydrophone (polyvinylidene fluoride, Precision Acoustics, Dorchester, UK) was used to measure Doppler beam width at the focal position for each of the transducers. The pressure pulse was captured by a digital oscilloscope (TDS2024 B, Tektronix, Beaverton, OR, USA). The hydrophone system had previously been calibrated in combination with a submersible preamplifier, a direct-current coupler and a 50-Ω “in-line” shunt up to 60 MHz, by the National Physical Laboratory (Teddington, UK). During measurement, the active aperture of the hydrophone was placed on a 3-D-positioning system (VisualSonics). The positioning system included a bench-mounted adjustable rail system (*x-y* direction) together with an adjustable RMV transducer stand (*z* direction). The mechanical platform was able to move the hydrophone at 0.1-mm intervals.

The –3-dB beam width at the focal plane was determined by the distance between the points whose receiving pulse amplitude was 3 dB below the maximum value on the fitting curve of the beam profile. The beam profile was acquired by connecting the measured peak amplitude of the signal along the *y*-axis (symmetrically 5 points on either side of the position of the maximal signal at 0.1-mm step). It was assumed that the beam profile was symmetric in *x* and *y*.

### O-ring phantom

**Fig. 1:**
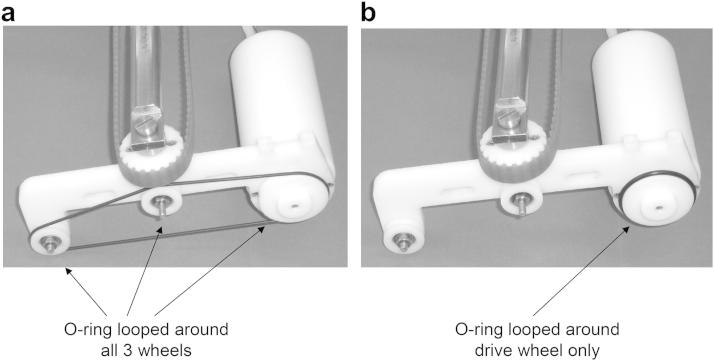
(a) String phantom in its original form with the O-ring looped around all three wheels. (b) Modified string phantom with the O-ring looped around the drive wheel only.

A phantom consisting of a modified string phantom was built. This was based on an existing string phantom (BBS Medical Electronics, Hägersten, Sweden). Preliminary work was undertaken with the string phantom used in its conventional format, with the O-ring rubber looped around the drive wheel and two free wheels (Fig. 1a). It was found that there was a vibration of 1 mm that could be clearly seen on the B-mode image, and a similar oscillation on spectral Doppler. To eliminate the string vibration, the O-ring was mounted on the drive wheel only (Fig. 1b).

The true speed of the O-ring was obtained using a stroboscopic technique. Regular flashes of light were generated by a light-emitting diode connected to a function generator. These were directed at a small mirror placed on the base of the drive wheel. The frequency of generation of light flashes was adjusted so that it matched the rotational frequency of the drive wheel. The required strobe frequency was set so that the mirror appeared frozen in position on the drive wheel. The velocity of the moving string is then given by

(7) $$V\left(\max,measured\right)=2\pi rf_{r}$$

where *r* = radius of the rotation of the string (1.8 cm).

### Rotating phantom

A rotating phantom composed of tissue-mimicking material (TMM) was manufactured. The recipe for the TMM was developed for use with clinical ultrasound systems (Teirlinck et al. 1998). Recently, the TMM has been found to have acoustic properties suitable for use in high-frequency ultrasound systems including preclinical scanners (Sun et al. 2012). Rotating TMM phantoms have been widely used in testing of clinical Doppler ultrasound and tissue Doppler imaging (TDI) systems (reviewed in Hoskins 2008). Different target velocities and beam-target angles can be obtained by placing the sample volume at different positions within the phantom. Preliminary work using a large-diameter rotating phantom, as would be used for evaluation of clinical ultrasound systems, was undertaken using the preclinical system. However, it was found that data from only a very restricted set of beam-target angles could be obtained. The methods below describe the development of a miniature rotating TMM phantom suitable for use with preclinical ultrasound systems.

**Fig. 2:**
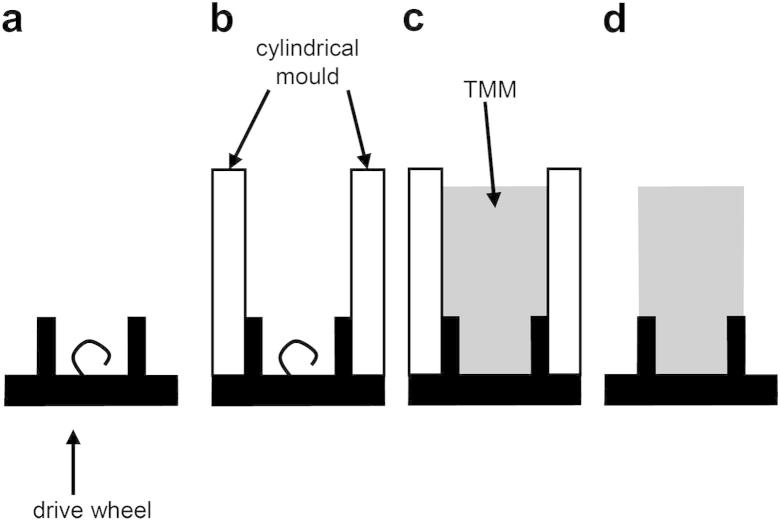
Construction of the rotating tissue-mimicking material (TMM) phantom. (a) Side view of the drive wheel showing the loop of wire used to secure the TMM. (b) A cylindrical mold is attached to the drive wheel. (c) Tissue mimic is poured in and allowed to set. (d) The mold is removed.

**Fig. 3:**
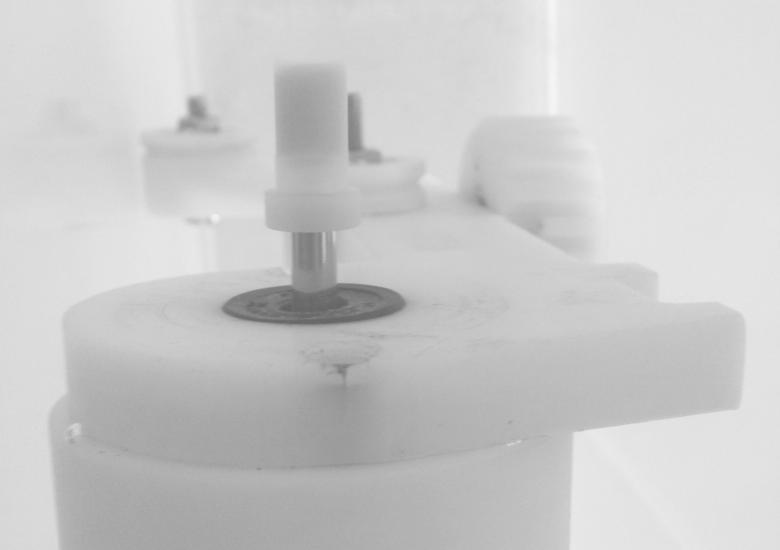
Final rotating tissue-mimicking material (TMM) phantom, showing the TMM and drive wheel attached to the motor by a steel shaft.

An existing string phantom (BBS Medical Electronics) was modified to provide rotation of the TMM phantom. The diameter of the phantom was chosen as 6 mm to visualize this within the field of view of all five transducers. A new drive wheel was manufactured to support the TMM. During preparation of the phantom, the new drive wheel was separated from the motor. Figure 2a shows the new drive wheel. A mold was created by attaching a clear plastic cylinder to the drive wheel (Fig. 2b). Tissue-mimicking material was poured into the mold and allowed to set. Adhesion of the TMM to the drive wheel was enabled through the use of a projecting loop of wire, as indicated in Figure 2(a, b). Once the TMM had set, the mold was removed and the drive wheel with TMM was attached to the motor. The final rotating TMM phantom is shown in Figure 3. For storage, the phantom was submerged in a 9% glycerol solution by volume.

The size of the phantom enabled the beam-target angle to be altered in the range 10°–80° for each transducer (except RMV 708).

The phantom was held by a retort stand and submerged in a tank filled with 9% (by volume) glycerol solution, which has an acoustic velocity of 1540 ms^−1^ at 20°C (Hoskins 2008). An acoustic absorber pad was placed at the bottom of the tank to reduce ultrasound reflections. The direct-current motor was driven by a controller (BBS Medical Electronics), which could be used to adjust the rotational speed of the phantom.

To measure the true linear velocity of the rotating TMM, a tiny dent was made on its surface that gives a periodic spike on the pulsed wave Doppler spectrum. The velocity of the rotating TMM at its edge is given by

(8) $$V_{\left(\max,\text{true}\right)}=\pi d/T$$

where *d* = diameter of the TMM (6 mm); and *T* = period of the spikes.

### Acquisition of spectral Doppler data

The phantoms were positioned on an *x-y-z* table (VisualSonics). The velocity of the rotating TMM (at the maximum diameter) and of the O-ring was set to a true value of 20 cm s^−1^, and spectral data were acquired with the sample volume placed on the surface of each phantom. For the O-ring phantom, it was necessary to attenuate the Doppler signal strength using a thin layer of TMM placed between the transducer and the phantom. All measurements were made with the Doppler gate positioned at the focus (Table 1). The gate length and center frequency for each transducer are given in Table 2. The phantoms were repositioned to obtain a range of beam-target angles, ensuring that the sample volume was located at the beam focus in each case. For the rotating phantom, Doppler data were acquired with a range of beam-target angles from 10° to 80°. For the O–ring phantom, a more restricted range of angles was possible because of the diameter of the drive wheel (35 mm). In practice, measurements were only taken on the RMV 710B transducer, which had the longest focal length. This allowed an angle down to 30° to be set without the O-ring rubbing against the transducer face.

**Fig. 4:**
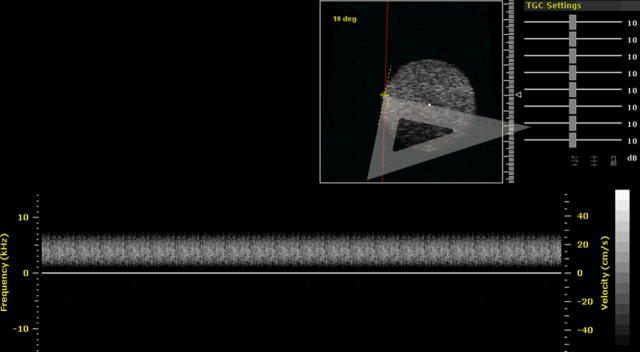
B-mode image and Doppler spectrum of the rotating tissue-mimicking material (TMM) phantom using RMV 710B. A set square was used to determine the tangent of the sample gate at the edge. The round bright area is the B-mode image of the rotating TMM phantom; a red line denotes the ultrasound beam. The angle correction cursor was placed on the edge of the TMM phantom. The current angle is 10°. The bottom spectrum is the Doppler spectrum of the rotating TMM.

The angle correction cursor was aligned with the direction of motion, and Doppler data were acquired. The Doppler gain was adjusted so that the spectral Doppler signals used the full gray scale available on the display. This procedure involved an increase in gain values until a few pixels reached peak white on the display. Note that a systematic procedure for setting of gain is important as estimated maximum velocity is dependent on Doppler gain (Hoskins et al. 2010). In Figure 4 are a B-mode image and a typical Doppler spectrum for the rotating TMM phantom at a beam-target angle of 10°. The beam-target angle was set by moving the sample gate to the edge of the rotating TMM. A set square was used to determine the tangent of the sample gate at the edge. The angle correction cursor was adjusted to align to the bottom edge of the set square, as indicated in Figure 4.

The Doppler digital IQ data were transferred off-line, and spectral data were reconstructed using software provided by the manufacturer. The maximum Doppler frequency envelope on the spectral trace was found using an in-house program employing an adaptive threshold method (Hoskins and McDicken 1991). The values for pulse repetition frequency (PRF) (30–40 kHz), speed of sound (1540 ms^−1^), transmit frequency (20–40 MHz) and beam-target angle (10°–80°), as displayed on the ultrasound system display screen, were imported into the off-line program to calculate the maximum velocity.

## Results

### Beam widths

Table 3 lists the 3-dB beam widths of the five transducers at the focus. The beam widths are similar and in the range 0.14–0.17 mm.

### Measured error with angle

**Fig. 5:**
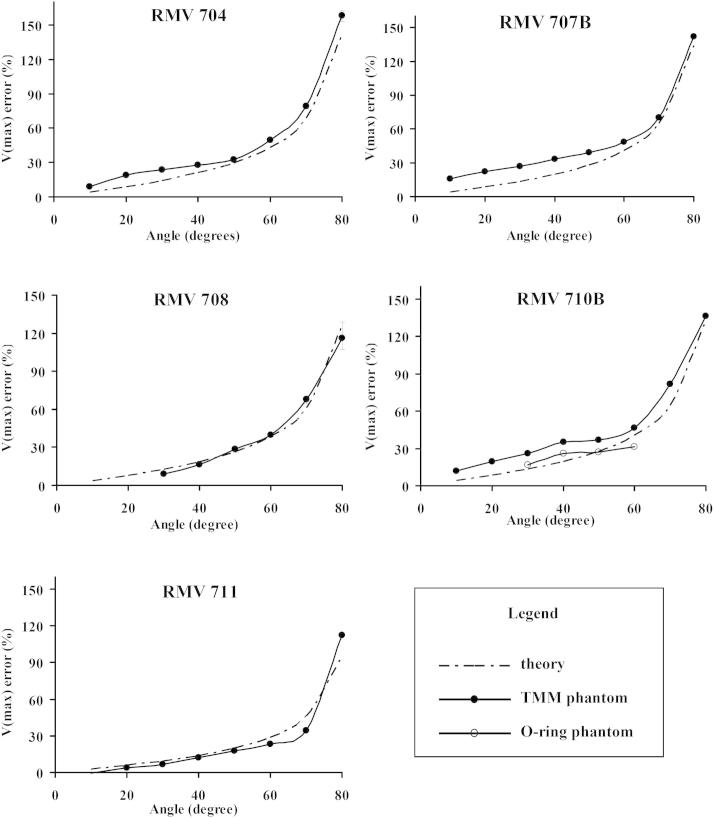
Maximum velocity error as a function of beam-target angle for the five RMV transducers. Experimental results obtained using the rotating tissue-mimicking material (TMM) phantom are compared with the error theoretically calculated using the zero-beam-width model. In addition, for transducer 710B, there are data from the rotating O-ring phantom.

Figure 5 illustrates the Doppler estimated maximum velocity error for the five RMV transducers. The figures show the velocity error measured using the rotating TMM phantom compared with the theoretical error made using the zero-beam-width model. In each case, the error increases with angle from 20° to 80°. The results for the rotating O-ring phantom obtained from the 710B transducer are also shown.

Table 4 outlines the difference between theory and experiment (rotating phantom) for maximum velocity, averaged across all transducers, and divided into angles 10°–40° and 50°–80°. There is little difference between theory and experiment for finite-beam-width and zero-beam-width models. Errors were low (<10%) for angles of 50°–80°, but much higher for angles below 50°. Table 5 shows the mean measurement errors among the five transducers with the beam-target angle from 10° to 80°.

## Discussion

In this study, the maximum velocity was calculated using off-line software rather than the on-board calculation package available on the ultrasound machine. Off-line software was used primarily to ensure consistency between measurements (using an automated procedure for estimation of velocity). In addition, the work was part of a wider project concerned with measurement of quantities related to velocity, such as flow rate and wall shear rate, which required off-line processing. Initial checks had been made that revealed consistency between maximum velocity estimated using the machine and that estimated off-line.

The increase in velocity error with angle is consistent, with the error arising from geometric spectral broadening. The velocity errors, up to 158% at the beam-target angle of 80°, are similar to those from clinical systems (though it is noted that most clinical systems use linear arrays, not single-element transducers).

The ray model of geometric spectral broadening gave good agreement with experimental results acquired from the rotating phantom (<10%) for angles in the range 50°–80°. However, agreement was poor for smaller angles, with a discrepancy of >50% for angles of 10°–40°. Geometric spectral broadening increases as angle increases. The good agreement at high angles suggests that geometric spectral broadening is the dominant cause of velocity error for angles in the range 50°–80°. However, for smaller angles, where geometric spectral broadening is smaller, it is possible that other sources of error may be more relevant. Further clarification of the source of the error would require the use of a more complex model of the Doppler measurement process.

There was little difference between the zero- and finite-beam-width models for the maximum frequency error, as indicated in Table 3. The finite beam width contributes a 0.5%–2% difference in maximum frequency over the zero-beam-width case, for typical depths, aperture positions and beam-vector angles *θ*.

Because of the rotation of the phantoms, there will be a range of target directions within the sample volume. This could act as an unwanted source of spectral broadening. It is noted that the focal width is typically 0.15 mm. For a sample volume of the same width, this subtends an angle of 0.5° for the O-ring phantom. This corresponds to spectral broadening of 0.3% and 1.4% at beam-target angles of 20° and 60°, respectively. If it is assumed that broadening is symmetric, the corresponding overestimation of maximum velocity will be half these values, that is, 0.15%–0.7%. These values are small compared with the velocity errors, and provide support for the use of the string phantom as a test tool. However, because of the size of this phantom, only a small range of beam-target angles could be evaluated. On the other hand, similar calculations for the rotating TMM phantom give values of spectral broadening of 1.9% and 8.7% at beam-target angles of 20° and 60°, respectively. This corresponds to an overestimation of maximum velocity of 1%–4%. The small size of the rotating TMM phantom has made it possible to obtain data over a very wide range of beam-target angles; however, this has been at the expense of a slight overestimation of the velocity error. Use of a belt phantom may provide improved results; however, this would be more difficult to manufacture compared with a rotating phantom.

The measured velocity errors for the two phantoms followed a similar overall trend between 30° and 60° on RMV 710B, but the average numerical values differed by 30.1% (the velocity errors measured using the rotating TMM phantom were generally higher than those measured using the O-ring phantom). There are several possible explanations for the difference in velocity error. First, as noted earlier, there is spectral broadening as a result of a range of target directions, and this effect is more pronounced for the smaller-diameter rotating TMM phantom, accounting for some 1%–4% of the difference in estimated maximum velocity for angles of 20°–60°. Second, the backscatter from the O-ring was considerably higher than that from the tissue mimic. It is possible that the reduction in Doppler gain plus the possible effects on beam width caused by the attenuator resulted in changes in beam geometry and observed Doppler frequency shift. The second possible explanation is that the backscatter directivity function differs between the O-ring and the tissue mimic. Directional peaks in backscatter are known to be a feature of thread-based string phantoms (Cathignol et al. 1994; Hoskins 1994). These manifest themselves as prominent horizontal bands on the Doppler spectrum. No such bands were seen on the Doppler spectra for either phantom in this study. However, there may be more subtle differences in backscatter directivity between tissue mimic and O-ring at the frequencies used in this study that warrant further investigation in a future study. For future use, it is recommended that the rotating phantom be used.

The current ultrasound system consisted of single-element mechanically swept transducers. Further work needs to be performed to evaluate errors in array transducers.

## Conclusions

Prototype phantoms have been developed for the validation of Doppler estimated blood velocity applicable to preclinical ultrasound scanners. The phantoms produced stable Doppler sources. Initial results indicated overestimation of maximum velocity by 1% to 158% by spectral Doppler. The increase in velocity error with beam-target angle is consistent with the source of error arising from geometric spectral broadening. The beam widths at the focal plane were also investigated to create the predicted errors from the ray model of geometric spectral broadening, with both the zero- and finite-beam-width assumptions. The rotating TMM phantom is capable of validating the performance of blood velocity measurement in preclinical ultrasound scanners.

## Figures and Tables

**Table 1:** Parameters of the five RMV scan heads

Model	Applications	Focal length (mm)	Active aperture size (mm)	Field of view (mm)	Axial resolution (μm)	Lateral resolution (μm)
704	Mouse vascular imaging Small mouse cardiac Mouse superficial embryonic Mouse abdominal	6	3	14.5	40	80
707B	High frame rate Mouse cardiac Mouse EKV	12.7	6	16.5	55	115
708	Mouse epidermal Skin cancers Bowel imaging Peritoneum	4.5	2	10.9	30	70
710B	High frame rate Rat cardiac Rat EKV	15	7	20	70	140
711	Guided injection, Superficial embryonic injection	6	2	8.4	30	90

EKV = electrocardiography-gated kilohertz visualization.

**Table 2:** Gate length and center frequency for the five RMV transducers

Model	Gate length (mm)	Center frequency (MHz)
704	0.05	30
707B	0.07	23
708	0.04	40
710B	0.08	20
711	0.04	40

**Table 3:** Beam widths of the five transducers

Model	Beam width (mm)
704	0.17
707B	0.14
708	0.15
710B	0.15
711	0.14

**Table 4:** Difference between measured (rotating phantom) and predicted errors in maximum velocity

	10°–40°	50°–80°
Mean difference between measured and zero beam width-predicted errors	55%	9%
Mean difference between measured and finite beam width-predicted errors	58%	10%

**Table 5:** Mean measurement errors in percentage (%±SD)

	RMV 704	RMV 707	RMV 708	RMV 710B	RMV 711
10°	8.8 ± 0.1	15.7 ± 2.0	—	12 ± 0.6	−0.5 ± 0.2
20°	18.7 ± 0.7	21.9 ± 0.7	—	19.3 ± 1.1	3.9 ± 0.2
30°	23.5 ± 0.5	26.7 ± 1.3	8.9 ± 0.5	26.1 ± 0.8	6.6 ± 0.4
40°	27.5 ± 0.7	33.3 ± 1.5	16.4 ± 0.1	34.9 ± 1.2	12.2 ± 0.1
50°	32.7 ± 1.6	39.1 ± 0.7	28.5 ± 2.0	37.0 ± 0.9	17.7 ± 0.4
60°	49.3 ± 1.4	48.7 ± 1.2	39.7 ± 1.6	46.7 ± 1.1	23.2 ± 0.3
70°	79.2 ± 2.3	70.0 ± 0.7	67.9 ± 2.5	81.8 ± 1.6	34.3 ± 0.1
80°	158.1 ± 4.0	141.7 ± 09	116.3 ± 9.2	136.0 ± 0.6	112.7 ± 0.2
